# RELIABILITY AND VALIDITY OF A SHORT VERSION OF THE NORWEGIAN AWARENESS OF SOCIAL INFERENCE TEST: A TEST OF SOCIAL COGNITION AFTER TRAUMATIC BRAIN INJURY

**DOI:** 10.2340/jrm.v58.46403

**Published:** 2026-10-06

**Authors:** Martin MATRE, Truls JOHANSEN, Anne Catrine T. MARTINSEN, Alexander OLSEN, Sveinung TORNÅS, Cecilie RØE, Anne LUND, Frank BECKER, Cathrine BRUNBORG, Daniel LØKE, Jacoba M. SPIKMAN, Jennie PONSFORD, Dawn NEUMANN, Skye MCDONALD, Marianne LØVSTAD

**Affiliations:** 1 Centre for Research and Education, Sunnaas Rehabilitation Hospital, Nesodden; 2 Department of Psychology, Faculty of Social Sciences, University of Oslo, Oslo; 3 Department of Occupational Therapy, Institute of Rehabilitation Science and Health Technology, Faculty of Health Sciences Oslo Metropolitan University, Oslo; 4 Institute of Rehabilitation Science and Health Technology, Oslo Metropolitan University, Oslo; 5 Department of Psychology, Norwegian University of Science and Technology, Trondheim; 6 Clinic of Rehabilitation, St. Olavs Hospital, Trondheim University Hospital, Trondheim; 7 NorHead, Norwegian Centre for Headache Research, Trondheim; 8 The Norwegian Directorate of Health, Oslo; 9 Institute of Clinical Medicine, University of Oslo, Oslo; 10 Department of Physical Rehabilitation and Medicine, Oslo University Hospital, Oslo; 11 Oslo Centre for Biostatistics and Epidemiology, Oslo University Hospital, Oslo, Norway; 12 Department of Neurology, Subdepartment of Neuropsychology, University of Groningen, University Medical Center, Groningen, The Netherlands; 13 Turner Institute for Brain and Mental Health, School of Psychological Sciences, Monash University, Clayton, Australia; 14 School of Psychological Sciences, Epworth Healthcare, Richmond, Australia; 15 Department of Physical Medicine and Rehabilitation, JFK Johnson Rehabilitation Institute, Edison, NJ, USA; 16 School of Psychology, University of New South Wales, Kensington, Australia

**Keywords:** social cognition, traumatic brain injury, rehabilitation, validation study

## Abstract

**Objective:**

This study aimed to develop and test reliability and validity of a short version of the Norwegian Awareness of Social Inference Test to ease assessment of social cognition after traumatic brain injury.

**Design:**

The least suitable items were identified statistically and from quality ratings made by clinical experts and persons with lived experience. Psychometric properties of the resulting short version were then estimated.

**Subjects:**

Adults with (*n* = 101) and without (*n* = 50) traumatic brain injury.

**Methods:**

Predetermined fit criteria from Rasch and confirmatory factor analysis identified least-fitting items. Further item reduction was based on expert ratings. The reliability of the short version was estimated with Cronbach’s alpha for internal consistency and intraclass correlations for test–retest reliability. Construct validity was estimated based on differences in performance between individuals with and without traumatic brain injury (known groups validity), correlations with measures of social cognition (convergent validity) and with measures of cognitive ability (discriminant validity).

**Results:**

The number of items was reduced from 59 to 28. Statistical analyses supported the short version’s structure, and reliability and validity were similar to the full version.

**Conclusion:**

The short version has acceptable psychometric properties and is more feasible than the full version; its implementation is recommended.

## LAY ABSTRACT

Relationship problems and social isolation are major challenges after traumatic brain injuries. One reason is impaired ability to take other people’s perspective. Despite this, such impairments are seldom assessed in brain injury rehabilitation. A major reason for this is a lack of good tests, especially outside the English-speaking world. The Norwegian Awareness of Social Inference test was recently developed to rectify this problem. However, its lengthy administration time is a barrier to clinical use. This study used statistical methods and expert ratings by clinical experts and people with lived experience of traumatic brain injury to reduce the number of items in the full version of the test by half. The result is a short version that can identify impaired ability to take other people’s perspective, which will ease clinical use. This will help rehabilitation professional address the social problems often experienced by individuals with traumatic brain injury.

Social cognition is an umbrella term for the different functions required to perceive, interpret, and interact with emotions, feelings, and intentions of others (1). Social cognitive impairments after traumatic brain injury (TBI) are common and may have debilitating consequences, such as reduced social participation (2) and relationship difficulties (3). The Awareness of Social Inference Test (TASIT) uses video-based tasks to assess two social cognitive functions: emotion recognition and Theory of Mind in individuals with TBI. Emotion recognition is a low-level process involved in the recognition of emotions of others, while Theory of Mind is the higher-level ability to think about one’s own and other people’s mental states (1). TASIT has demonstrated adequate test–retest reliability (0.74–0.88) and construct validity in that it is associated with other tests of social cognition and discriminates between individuals with and without TBI (known groups validity) (4, 5). A recent international survey of 260 clinicians found that TASIT was the most commonly used test of social cognition (6). As its lengthy administration was a barrier to adoption in research and clinical settings, a short version was developed (7).

To make TASIT available to non-English speaking populations, it has been adapted to a number of languages, including Danish (8), Dutch (9), German (10, 11), and Spanish (12). However, there is a lack of validated Norwegian tests of social cognition after TBI. This is a problem, as standardized assessment is an important prerequisite for successful rehabilitation after TBI. For this reason, a Norwegian version of TASIT (N-TASIT) has recently been developed (13), and has been demonstrated to have adequate reliability and construct validity (14). In order to explore virtual reality (VR) technology’s potential for ecological validity, both a 2D and a VR version of N-TASIT have been developed. Both versions have been found to be associated with everyday social functioning (15). However, the administration time of 60 min makes assessment strenuous for patients and research subjects, and overly time-consuming for clinicians and researchers. A short version would ease N-TASIT’s adoption clinically and in research, reducing demand on those tested, as well as saving time and money. This study’s aims were therefore to establish a short version of N-TASIT and determine its reliability and validity.

## METHODS

This study complies with the COSMIN Reporting Guideline for studies on measurement properties, except for elements pertaining only to patient-reported outcomes, as N-TASIT is a performance-based measure (16). Establishment of the N-TASIT short version was based on the same sample and used the same dataset as that used in development of the full N-TASIT. It included 101 individuals with TBI and 50 non-TBI participants matched on age, sex, and education. Table I describes participants’ demographic and injury related characteristics. The study had an observational cross-sectional design, with all participants being assessed with measures of convergent and divergent validity at time point 1 (T1) and then 16 weeks later at time point 2 (T2) for test–retest reliability analysis. Individuals with TBI were recruited and randomized to perform N-TASIT in either 2D or VR presentation mode. The initial target was for the 2 groups to be equally sized. However, due to the research project’s timeline constraints, and slow recruitment of non-TBI persons, it was decided to settle for 50 non-TBI participants. As a consequence, the majority of non-TBI participants were allocated to the VR group to secure enough power for comparisons to be made in this condition. Blinding was not possible due to the nature of the two conditions. To establish known group validity, i.e., that a measure differentiates between individuals with and without social cognitive impairments, N-TASIT performance in the TBI group was compared with that of the non-TBI group.

**Table I:** Participant demographics and injury characteristics

Characteristic	TBI (*n*=101)	non-TBI(*n*=50)	*p*-value
Age (mean, SD)	45.8 (13.8)	44.5 (13.4)	0.669
Sex, *n* (percentage)			0.518
Male	76 (75.2%)	40 (80%)	
Female	25 (24.8%)	10 (20%)	
Years of education, mean (SD)	13.6 (2.8)	14.8 (2.1)	0.005
Injury severity, *n* (percentage)			
Complicated Mild	22 (21.8%)		
Moderate	12 (11.9%)		
Severe	67 (66.3%)		
Years since injury, mean (SD)	5.5 (3.2)		

*p*: probability; SD: standard deviation.

Participants in the TBI group were identified from medical journals of former patients with TBI at Sunnaas Rehabilitation Hospital and invited to participate. Semi-standardized screening interviews were conducted to verify that those who consented to participation met inclusion criteria. Non-TBI participants were recruited through advertisement on social media. Participants with TBI were asked to invite a close friend or relative to provide information concerning everyday functioning. Data were collected between September 2022 and May 2024.

Individuals with TBI were recruited according to the following inclusion criteria:

- A radiologically verified complicated mild-to-severe TBI, with severity of TBI being classified by a combination of the Glasgow Coma Scale (17), loss of consciousness, and duration of post-traumatic amnesia (18). Distinctions between mild and complicated mild TBI were based on the criteria of the American Congress of Rehabilitation Medicine, i.e., that the qualifier “complicated” is used to denote neuroimaging evidence of structural intracranial injury (19).
- Between 1 and 10 years post-injury.
- Aged 18–65 years old.
- Able to provide informed consent.

Exclusion criteria were:

- Severe aphasia or apraxia affecting the ability to understand instructions or operate VR equipment.
- Hemianopsia or visual neglect.
- Severe mental illness (e.g., schizophrenia, bipolar disorder, or severe depression) or alcohol/substance abuse.
- Comorbid neurologic or neuropsychiatric disorder.
- Aged < 16 at the time of injury.
- Non-Western background.

Inclusion and exclusion criteria for the non-TBI group were identical to those for the TBI participants, except for TBI being an exclusion criterion instead of an inclusion criterion.

### Ethics and data security

The study was undertaken in accordance with the Declaration of Helsinki and approved by the Regional Committee for Medical Research Ethics (reg.nr. 376999) and the data protection agency SIKT (Norwegian Agency for Shared Services in Education and Research; reg.nr. 172224). The study protocol was preregistered in Clinical Trials (NCT05309005) and Open Science Framework (osf.io/2vem8). Participants provided written informed consent. Questionnaire data were collected and stored by digital systems hosted by the University of Oslo (Nettskjema/Service for Sensitive Data, TSD).

### Measures

N-TASIT is a Norwegian version of the original Australian TASIT (4, 5), which assesses emotion recognition and Theory of Mind using short video vignettes. It is criterion-based, with modest difficulty for individuals without social cognitive impairment. N-TASIT has high resemblance to the original in both form and content. Both a Virtual Reality and a 2D version were developed. See Matre et al. (13) for a detailed description of test development.

Like the original TASIT, N-TASIT consists of 3 parts. Part 1 measures recognition of 7 emotional states (anger, sadness, fear, disgust, surprise, happiness, neutral), with each emotion being presented in 4 videos, resulting in a maximum total score of 28. Parts 2 and 3 measure Theory of Mind. In Part 2, one of the actors is either sarcastic or sincere and the task is to infer if what is said was literally meant. Participants respond to 4 yes/no questions in each of the 15 videos, pertaining to the beliefs, intentions, behaviour, and emotional state of the characters, i.e., maximum 60 points. In Part 3, 1 of the actors is either lying or being sarcastic in a setting with more context than in Part 2, followed by 4 questions in each of the 16 videos, giving a maximum of 64 points.

Previous publications have demonstrated that N-TASIT has adequate reliability, construct validity (14), and ecological validity (15). Test–retest reliability of the total score was 0.59 for individuals with TBI, and Cronbach’s alpha was 0.87. All 3 parts differentiated between individuals with and without TBI, which is an indication of known groups validity. Convergent and divergent validity was manifested by association with other measures of social cognition and a relative lack of association with measures of other cognitive functions and emotional distress.

### Measures of convergent validity

The Hinting Task is a test of Theory of Mind where the task is to infer the subtext of a hint in 10 verbal scenarios (20). It has been translated into Norwegian (21) and shown to be sensitive to impairments after TBI (22). The Emotion Recognition Task (ERT) is a well-validated test where the task is to correctly label emotional facial expressions (23). The Interpersonal Reactivity Index (IRI) is a commonly used measure of self-reported empathy (24), where the affective component of empathy is measured with the Empathic Concern subscale and cognitive empathy on the Perspective Taking subscale.

### Measures of discriminant validity

To explore the relationship between N-TASIT scores and domains other than social cognition, measures of cognition, mental health, and fatigue were included. Mental efficiency, working memory, and abstraction were measured with the subtests Coding, Digit Span Backward and Sequencing, Matrices and Similarities from the Wechsler Adult Intelligence Scale – Fourth Edition (25). Mental flexibility and inhibitory control were measured with Trail Making Test 4 and Color Word Interference Test 3 from the Delis–Kaplan Executive Function System (26). Self-reported executive functioning was measured with the Behavior Rating Inventory of Executive Functioning – Adult (27). The global executive composite (GEC), and index scores for meta-cognition (MI) and behavioural regulation (BRI) were used. Fatigue was measured with the Fatigue Severity Scale (28), while depressive and anxiety symptoms were measured with the Patient Health Questionnaire (PHQ-9) and the Generalized Anxiety Disorder (GAD-7), respectively (29, 30).

### Analytical approach and statistics

Reduction of items to a short version was based on a combination of the statistical approaches applied in the development of the Australian S-TASIT and expert quality ratings. Reliability and validity analyses were conducted for the final short version. Statistics were conducted using R, using the eRm package for Rasch analysis (version 4.0.3, R Foundation for Statistical Computing, Vienna, Austria).

Except for known groups validity estimates, analyses were performed on participants with TBI only, as this is the clinically relevant population and scores are least likely to be restricted in range. For demographic data, differences in continuous variables between participants with and without TBI were tested with independent sample *t*-tests, while a χ^2^ test was used to explore sex differences. Presentation modes (VR/2D) were collapsed due to lack of significant differences in the full version (14).

### Item selection process

*Step 1.* For each N-TASIT Part, a Rasch model of the full version was performed to extract item infit and outfit, with values between 0.8 and 1.2 being considered optimal and values between 0.5 and 1.5 considered acceptable (31, 32). Person and item reliability were estimated. A principal component analysis (PCA) based on the Rasch model was examined to assess unidimensionality. Contrast values > 2 were considered an indication of an additional dimension. Point biserial correlations (PBC) were examined, aiming for values between 0.3 and 0.7. Items not performing according to criteria were eliminated and the process was repeated. When statistical analysis no longer differentiated between items, final item selection was based on the quality ratings.

For Parts 2 and 3 item scores range from 0–4. Category consistency in Rasch modelling requires that category threshold values increase in ascending order. However, inconsistency was found, and for all items except items 3 and 6 in Part 1 the modal score was either 3 or 4, indicating a pass/fail response pattern. Based on this, 0–2 scores were collapsed and also 3–4 scores. Thus, all Rasch models were dichotomous.

To identify items with optimal psychometric properties, and determine the fit and factor structure of the final models, confirmatory factor analysis (CFA) was conducted. To compensate for violation of normality in CFA, the Bollen–Stine bootstrapping procedure was used. As for the original short version, fit values were considered ideal if normed χ^2^ value (χ^2^/df ) < 3, root mean square error of approximation (RMSEA) < 0.05 for the lower and < 0.10 for the upper bounds limits, comparative fit index (CFI) < 0.90, and standardized root mean square (SRMR) residual value < 0.10 (33). Where PCA indicated more than 1 dimension, CFA was also used to identify which factor solution best fitted the data.

*Step 2.* To reach approximately half as many videos as in the full version, as in the Australian short version, further item reduction was based on expert quality ratings. A balanced number of emotion categories was aimed at for after the statistical procedure. Each of the 58 videos was independently evaluated by 3 of the authors (TJ, ML, ST), all of whom have extensive clinical experience with social cognitive impairment after TBI. Four individuals with moderate/severe TBI, but not social cognitive deficits, also assessed all videos. Before each video, the correct answer was read out. Raters were then asked to rate how clearly and convincingly either an emotional display (Part 1) or the display of sarcasm/sincerity (Part 2 and 3) was enacted. Acting performance was rated on a 1–10 scale, from minimally (1) to maximally (10) clear and convincing. An average score was created for each item (see Tables SI–SIII for ranked lists for each part). Once the statistical approach described in step 1 no longer differentiated between items, the poorest ranking items that were left were chosen for removal.

*Step 3*. A final Rasch analysis was conducted on the items in the final short version. Separate analyses were conducted on parts with more than 1 dimension.

### Validation of short version

Test–retest reliability was assessed with intraclass correlation coefficients (ICCs; two-way mixed, absolute agreement, average measures) for Parts 1–3 and total scores. An ICC < 0.50 is indicative of poor reliability, 0.50–0.75 moderate, 0.75–0.90 good, and > 0.90 excellent reliability (34).

Internal consistency for Parts 1–3 and total scores were explored with Cronbach’s alpha. Alpha levels above 0.70 are generally considered acceptable, while levels above 0.90 are potentially indicative of redundant items (35).

Mann–Whitney *U* tests were used to explore known group validity and Spearman Rank Order coefficients were used to explore convergent and discriminant validity. The magnitude of effect size estimates for non-parametric tests was calculated as Pearson’s *r* (36), with Cohen’s guideline for classification: large = 0.50, medium = 0.30, and small = 0.10. Cohen’s guideline for classification of correlations was applied for exploration of convergent and discriminant validity: large = 0.50, medium = 0.30 and small = 0.20 (37).

## RESULTS

### Item selection

*Part 1.* Item 9 (Disgust) was discarded before statistical analyses due to low hit rate in both TBI (55%) and non-TBI (52%) groups. The first contrast value from PCA was 1.37, indicating only 1 underlying dimension. In the first Rasch model, only item 6 (Neutral) had infit or outfit values outside the targeted range, with an item outfit value of 0.47. As all items except items 6 and 13 (both Neutral) had PBCs below the 0.30 threshold, the 4 items with both a PBC value and factor loading from CFA ≤ 0.10 were removed. For a detailed description of the statistically driven item selection, see Tables SIV–SX. New analyses were then performed on the remaining 21 items, with a combination of factor loadings and PBC values guiding further item selection in addition to the Rasch criteria. When this procedure was repeated 6 times, 12 items remained and further item removal no longer improved either CFA or the Rasch model.

After the statistical procedure, items 4 (Disgust) and 10 (Sad) were removed due to poor quality scores (see Table SI for complete rankings). The final short version of Part 1 consisted of 10 items: Sad: item 5; Anger: items 12 and 23; Disgust; items 15 and 22; Fear: items 14 and 25; Happy; item 17; and Neutral; items 13 and 19.

A Rasch model with these items had infit and outfit values well within the pre-set criteria, as well as good item and person reliability. Average PBC was 0.23, which is below the recommended 0.30. See Table II for details. CFA values are given in Table III. All indicators of model fit were within the criteria suggested by Honan and colleagues (7), except for SRMR, which was just above 0.10.

**Table II:** Summary of Rasch analyses

TASIT Part	Item infit Mean	Item Infit Range	Item Outfit Mean	Item Outfit Range	Point-Biserial Range	Item-reliability	Person reliability
Part 1	0.93 (0.11)	0.80–1.06	0.87 (0.26)	0.51–1.25	0.14–0.30	0.87	0.76
Part 2 – Sincere	0.93 (0.06)	0.85–0.98	0.89 (0.13)	0.70–0.97	0.45–0.72	0.82	0.62
Part 2 – Sarcasm	0.92 (0.10)	0.78–1.06	0.89 (0.20)	0.69–1.15	0.35–0.60	0.95	0.68
Part 3 – Lies	0.94 (0.20)	0.71–1.02	0.95 (0.23)	0.74–1.25	0.14–0.43	0.85	0.46
Part 3 – Sarcasm	0.92 (0.12)	0.71–1.02	0.91 (00.09)	0.82–1.06	0.25–0.37	0.93	0.51

TASIT: The Awareness of Social Inference Test. Standard deviations are shown in parentheses.

**Table III:** Goodness of fit indices for the confirmatory factor analyses

TASIT Part	Chi^2^/df	RMSEA	90% CI for RMSEA		CFI	SRMR
			Lower	Upper		
Part 1	0.96	0.00	0.00	0.07	1.00	0.17
Part 2 One factor	1.92	0.96	0.06	0.14	0.87	0.09
**Part 2 Two factor**	**1.27**	**0.05**	**0.00**	**0.10**	**0.96**	**0.07**
Part 3 One factor	2.67	0.13	0.09	0.17	0.66	0.12
**Part 3 Two factor**	**1.56**	**0.08**	**0.02**	**0.12**	**0.89**	**0.09**

χ^2^/df: normed chi-square; CFI: comparative fit index; CI: confidence interval; LB: lower bound; RSMEA: root square mean error of approximation; SRMR: standardized root mean square residual; TASIT: The Awareness of Social Inference Test; UB: upper bound. Text shown in bold is indicative of the better-fitting models.

As in the Australian version, all Surprise items were eliminated in the statistical process. Based on findings in previous research (23), *post hoc* analyses were performed to explore systematic misclassification of Surprise items. It was found that of the 74 erroneously categorized Surprise responses, 60 (80.1%) were labelled Happy, which is more than expected in a random distribution.

*Part 2.* The first contrast value from PCA of Part 2 was 2.77, indicating more than 1 underlying dimension. Second contrast value was 0.41. The CFA indicated that a 2-factor solution with sincere and sarcastic items as separate dimensions was a better fit than a 1-factor structure (Δχ^2^ = 6.01, df = 1, *p* = 0.014). Item selection was thus conducted with these 2 factors separately. All Sincere items had item infit/outfit values from the Rasch model within acceptable range. Item 7 had PBC < 0.3 and was therefore removed. Sarcasm items 8 and 6 were removed due to outfit values not meeting criteria. All sarcasm items had PBC values within ideal range of 0.3–0.7, and infit/outfit values within range. Item 7 had PBC ≤ 0.3 and was therefore removed (results in Tables SXI–SXIV). Finally, items 10 and 13 were removed based on poor quality scores (see Table SII). The following 0 items were selected for Part 2: 1, 2, 3, 4, 5, 9, 11, 14, and 15. Of these, 5 were Sarcasm and 4 were Sincere items, which is identical to the Australian version. The Rasch model had acceptable fit values for both Sarcasm and Sincerity items, as was the case for CFA (see Tables II and III).

*Part 3.* Rasch PCA of the residuals found a first contrast value of 2.04 and second contrast value of 0.90, indicating more than 1 underlying dimension. A 2-factor solution of the full version, with Lies and Sarcasm as separate factors, fitted the data better than a 1-factor structure. (Δχ^2^ = 21.71, df = 1, *p* < 0.001). Items were therefore selected separately for Sarcasm and Lies items.

Two Lie items (7 and 11) were removed as item outfit values extracted from the Rasch model were outside acceptable range. Of the Sarcasm items, only item 5 was below the PBC threshold of 0.3. All items were within desired thresholds in the next iteration of analyses (see Tables SXV–SXVIII). Items 1, 3, 4, 5, and 14 were therefore eliminated based on quality ratings (Table SIII details scores). This resulted in a total of 9 items in the final short version of Part 3 (2, 6, 8, 9, 10, 12, 13, 15, and 16), of which 5 were Sarcasm and 4 Lies items, as in the Australian short version.

A Rasch model suggested satisfactory fit values for both Sarcasm and Lie items (see Table II). The same was the case for CFA, except for the upper limit of the confidence interval of RMSEA, which was slightly above ideal range (see Table III for details).

### Validation of the short version

*Reliability.* Table IV presents reliability data for the N-TASIT short version compared with that of the full version. Cronbach’s alpha for the Total score was slightly lower in the short version compared with the full version, but at an acceptable level (α = 0.71 vs α = 0.87). The short version’s Total score had markedly better ICC relative to the full version (0.85 vs 0.59), while Part 1 ICC was poorer (0.57 vs 0.75) in the short version compared with the full version, and Parts 2 and 3 were very similar to the full version.

**Table IV:** Reliability of the short version vs the full version of N-TASIT for participants with TBI

TASIT Part	Short version			Full version		
	Cronbach’s alpha	ICC	95% CI	Cronbach’s alpha	ICC	95% CI
Total	0.71	0.85	0.77–0.90	0.87	0.59	0.40–0.72
Part 1	0.54	0.57	0.34–0.72	0.55	0.75	0.64–0.83
Part 2 – Sincere	0.68	0.86	0.79–0.91	0.60	0.71	0.55–0.81
Part 2 – Sarcasm	0.76	0.86	0.79–0.91	0.83	0.85	0.76–0.90
Part 3 – Lie	0.68	0.85	0.78–0.90	0.76	0.83	0.74–0.89
Part 3 – Sarcasm	0.59	0.83	0.74–0.89	0.71	0.88	0.82–0.92

ICC: intraclass correlation coefficient; 95% CI: 95% confidence interval; TASIT: The Awareness of Social Inference Test.

*Validity.* Known groups validity is displayed in Table V. Participants with TBI performed significantly more poorly than non-injured participants for Total scores and on each of the 3 parts, with effect sizes comparable to the full version (*r* = 0.36 vs 0.41 for Total scores).

**Table V:** Known group validity in the short version vs the full version of N-TASIT

TASIT Part	Group	Short version				Full version			
		Median	IQR	*r*	*p*-value	Median	IQR	*r*	*p*-value
Total	TBI	63.00	11.00	0.36	< 0.001	119.00	19.00	0.41	< 0.001
(max = 82)	non-TBI	70.00	7.00			131.00	14.00		
Part 1	TBI	9.00	2.00	0.26	0.002	23.00	4.00	0.41	< 0.001
(max = 10)	non-TBI	10.00	1.00			25.00	3.00		
Part 2	TBI	26.00	5.00	0.37	< 0.001	48.00	9.00	0.34	< 0.001
(max = 36)	non-TBI	29.00	4.00			53.00	6.00		
Part 3	TBI	28.00	5.00	0.26	0.001	48.00	9.00	0.30	< 0.001
(max = 36)	non-TBI	30.00	4.00			52.00	7.00		

IQR: interquartile range; *r*: effect size calculated as Pearson’s correlation coefficient; *p*: significance level; TASIT: The Awareness of Social Inference Test.

As in the full version, the ERT and Hinting Task, estimates of convergent validity, showed medium to large associations with the short version total score, and small to medium associations with all 3 parts. In contrast to the full version, correlations with self-reported perspective taking on IRI failed to reach significance. The short version total score was moderately associated with some measures of divergent validity (abstraction, working memory, processing speed, and executive functioning), but had non-significant associations with self-reported executive functioning, fatigue and mental distress (see Table SXIX for details).

## DISCUSSION

The present study demonstrated that the short version of N-TASIT (N-TASIT Short) had overall reliability and validity estimates comparable to the full version. The items selected for the N-TASIT Short fitted the Rasch model, with item infit and outfit levels within acceptable range (31). When the χ^2^ statistic was used as an estimation of CFA model fit, all parts were well within the recommended range. This was also the case for most other indices of fitness, with some minor exceptions, indicating that N-TASIT Short retained the full version’s characteristics. Crucially, N-TASIT Short differentiates between individuals with and without TBI, and the associations with both cognitive and social cognitive measures were comparable to those of the full version.

The fact that all but 1 item in Part 1 had point-biserial correlations (PBC) below the target value of 0.3 is probably a consequence of the limited range of scores, as PBC is sensitive to data variability. N-TASIT demands are intentionally light for participants without social cognitive impairment, with scores clustering towards the upper end of the distribution. This reduces correlations, affecting PBC.

In terms of Total score, N-TASIT Short had better test–retest reliability, but somewhat poorer internal consistency, than the full version. The latter finding is likely partly due to the reduction in number of items. Parts 2 and 3 of the short version also had marginally poorer internal consistency estimates compared with the full version, possibly as a result of dichotomizing items. Seen in isolation, these findings suggest that the full version is preferrable to the short version, at least for Parts 2 and 3. However, an inspection of the 2 Rasch reliability measures, which complement traditional reliability measures, nuances the picture. Person reliability in Rasch can be interpreted similarly to Cronbach’s alpha (38). As with PBC discussed earlier, both person reliability and Cronbach’s alpha are affected by the limited range of social cognitive ability measured by the test, accounting for why scores were either at or slightly below target levels in both the Norwegian and the Australian versions. In contrast, estimation of item reliability is dependent on variability in item difficulty. For this measure, the reliability scores were well above the acceptable level for all N-TASIT Short parts, indicating that they are fit for purpose.

Item 9 in Part 1 was removed prior to statistical analysis. This decision was made on the basis that both the TBI and the non-TBI group performed close to chance, suggesting that the actor did not display disgust in a clear enough manner. There was no predefined lower threshold for when to discard items prior to statistical analysis, but as the rest of the items were well above a hit rate at chance level, only this 1 item was excluded.

The results of the Rasch analysis were comparable to the original Australian short version TASIT, which used the same item reduction approach as the present study, except for the expert quality ratings. PBCs were somewhat poorer than in the original test. The convergent validity of the Norwegian Short version was slightly better than the Dutch version for the Total score for both emotion recognition and Theory of Mind, and superior in that all parts were significantly related to other tests of social cognition. Direct comparisons with the Danish, Spanish, and German versions are difficult, as they did not validate the tests on TBI samples.

### Expert quality ratings

The quality evaluations were useful in identifying items flagged as problematic by clinicians or people with lived experience. Perceived problems with video quality were mainly due to acting performances where an emotion was not clearly conveyed, or where attention was diverted to something irrelevant. Still, such considerations of face validity, i.e., indications of a measure’s “appropriateness, sensibility or relevance” (39), do not guarantee construct validity. Therefore, the quality judgements were a supplement to the psychometric analysis in the present study.

### The element of surprise

As the social scenes were enacted by different actors in the Norwegian and the Australian versions it was not expected that the same items would be retained. It was, however, interesting that as with the Australian short version, none of the surprise items conformed well to the Rasch and CFA models. Honan and colleagues (7) raised concerns with including Surprise in a test of emotion recognition. Among other reasons they note that surprise lacks a neurobiological correlate and positive or negative valence. Furthermore, surprise is often miscategorized as fear or happiness in experimental studies with non-impaired subjects (23). In the present study, both TBI and non-TBI participants were far more likely to misinterpret surprise as happiness than any other emotion. Taken together, the results support excluding surprise from N-TASIT Short.

### Limitations

Limitations related to the validation of the full version of N-TASIT necessarily carried over to the present study. Collapsing 2D and VR presentation mode may have introduced a confounding variable, in that watching VR may be more cognitively taxing than 2D. However, performance did not differ between modalities, which suggests otherwise. Also, the significant correlations with some cognitive measures indicate that the short version of N-TASIT is not a pure measure of social cognition. For instance, the dynamic and multi-modal nature of N-TASIT places demands on cognitive functions like processing speed and verbal comprehension. That said, it is a general problem that cognitive tests are rarely able to measure only 1 cognitive function in isolation.

As category consistency analysis found inconsistencies for most items in Parts 2 and 3, scores were collapsed into dichotomous scores. The loss of information this caused may have reduced the short versions’ sensitivity to different levels of social cognitive functioning, as well as affected the reliability and validity estimates. However, as category consistency is a prerequisite for Rasch analysis, it was a necessary decision.

Because the current sample included a higher proportion of participants with mild TBI than the Australian validation sample, and as the sample was unselected with regard to pre-established social cognitive problems, the data are probably more representative of the full range of TBI severity than those in the original test. On the other hand, the inclusion of persons with complicated mild TBI may have contributed to a lower proportion with social cognitive impairment. In addition, it may be argued that the sample size in the present study is small for both Rasch and confirmatory factor analysis. Small samples introduce a risk of imprecise estimates of model fit in confirmatory factor analysis. This risk is, however, reduced when the number of factors is low, as is the case here. Some argue that Rasch analysis with sample sizes < 200 risk reduced measurement precision (40). However, there is evidence that the conditional fit statistics incorporated in the R-package eRm provide robust and unbiased results with smaller samples (32). Finally, the validation of the short version used the same sample and dataset as the validation of the full version. This introduces a risk of overfitting and consequently overly optimistic estimates of the psychometric properties in the short version. Future studies should therefore be aim to replicate the current findings in other samples.

### Clinical implications and future directions

Cutting administration time in half will significantly increase the feasibility of administering N-TASIT, ensuring systematic assessment of social cognition clinically and in research. Future research should also provide a guideline for interpreting N-TASIT Short performance in terms of impairment.

This study did not establish whether N-TASIT Short is sensitive to change in social cognition. Responsiveness could be determined through calculation of minimally relevant change in future studies, to establish usefulness in clinical trials. It will also be important to determine whether the psychometric properties of N-TASIT Short are retained when examined in other populations, such as autism or schizophrenia.

In conclusion, the present study found that the N-TASIT Short overall has psychometric properties broadly in line with the full version, with only marginally poorer performance for some of the subscales. Consequently, the implementation of the short version is preferable both clinically and for research purposes in assessment of social cognitive impairments after TBI.

## Supplementary Material


