# Supplementary material for: RELIABILITY AND VALIDITY OF A SHORT VERSION OF THE NORWEGIAN AWARENESS OF SOCIAL INFERENCE TEST: A TEST OF SOCIAL COGNITION AFTER TRAUMATIC BRAIN INJURY
Source: J Rehabil Med. 2026 Oct 6;58:46403. doi: 10.2340/jrm.v58.46403 (PMC13650672; doi:10.2340/jrm.v58.46403)
Supplement: Supplementary file 1 [file JRM-58-46403-s1.pdf]

Supplementary Materials

Qualitative evaluations

Table SI: Ranking of items from Part 1 of N-TASIT, based on qualitative assessments

*Notes: TBI: traumatic brain injury. * Item 9 was discarded before statistical analyses due to low hit rate in both TBI (55%) and non-TBI (52%) groups.*

		Total			Clinical experts			Individuals with TBI		
Item nr	Emotion	Rank	Mean score	Standard deviation	Rank	Mean score	Standard deviation	Rank	Mean score	Standard deviation
1	Anger	12	7.6	2.1	17	5.7	1.2	8	9.0	1.2
2	Surprise	19	6.9	2.1	8	7.0	2.6	16	8.0	2.2
3	Happiness	22	6.4	2.4	6	7.3	1.5	24	5.8	2.9
4	Disgust	13	7.6	2.6	19	5.3	2.5	7	9.3	1.0
5	Sadness	16	7.0	3.1	11	6.7	2.5	20	7.3	3.8
6	Neutral	5	8.4	1.5	4	8.0	1.0	10	8.8	1.9
7	Fear	26	4.7	3.6	25	4.0	1.0	25	5.3	4.9
8	Happiness	3	8.6	1.5	7	7.3	1.2	6	9.5	1.0
9	Disgust*									
10	Sadness	17	7.0	2.2	13	6.3	2.1	19	7.5	2.4
11	Surprise	6	8.4	1.9	5	8.0	1.0	11	8.8	2.5
12	Anger	2	9.4	1.0	2	8.7	1.2	1	10.0	0.0
13	Neutral	8	7.9	2.7	21	5.0	0.0	2	10.0	0.0
14	Fear	24	5.1	3.2	22	5.0	1.0	26	5.3	4.4
15	Disgust	7	8.1	3.0	18	5.7	3.2	3	10.0	0.0
16	Sadness	4	8.6	1.0	3	8.7	0.6	13	8.5	1.3
17	Happiness	14	7.1	3.0	14	6.3	2.9	18	7.8	3.3
18	Anger	21	6.7	2.6	9	7.0	1.0	21	6.5	3.5
19	Neutral	15	7.1	3.1	15	6.0	1.0	17	8.0	4.0
20	Fear	27	4.4	3.4	27	2.3	0.6	23	6.0	3.9
21	Surprise	23	5.3	3.2	26	4.0	2.0	22	6.3	3.9
22	Disgust	9	7.9	3.0	20	5.3	3.2	5	9.8	0.5
23	Anger	1	9.7	0.5	1	9.3	0.6	4	10.0	0.0
24	Surprise	25	5.1	3.0	12	6.7	2.5	27	4.0	3.2
25	Fear	10	7.7	2.8	16	6.0	3.6	9	9.0	1.4
26	Happiness	11	7.7	2.4	10	7.0	2.6	15	8.3	2.4
27	Sadness	18	7.0	2.8	23	4.7	2.9	12	8.8	1.0
28	Neutral	20	6.9	2.5	24	4.7	1.2	14	8.5	1.7

Table SII: Ranking of items from Part 2 of N-TASIT, based on qualitative assessments

Note: TBI: traumatic brain injury.

		Total			Clinical experts			TBI individuals		
Item nr	Sarcasm/ sincere	Rank	Mean score	Standard deviation	Rank	Mean score	Standard deviation	Rank	Mean score	Standard deviation
1	Sincere	15	4.6	3.5	15	2.3	0.6	14	5.3	6.5
2	Sarcasm	5	7.9	2.9	2	7.7	0.6	7	3.3	3.1
3	Sarcasm	7	7.3	1.1	7	6.3	0.6	8	3.8	3.4
4	Sincere	1	8.4	1.1	1	8.0	0.0	4	2.5	3.7
5	Sarcasm	4	8.0	2.2	5	7.0	1.7	5	4.0	2.5
6	Sarcasm	8	7.3	2.8	10	5.0	2.0	3	5.0	3.6
7	Sincere	14	5.0	3.2	13	3.3	1.5	15	5.3	5.2
8	Sarcasm	3	8.3	2.4	8	6.3	2.5	1	4.8	2.8
9	Sarcasm	10	6.4	2.6	9	5.7	2.3	12	4.9	3.1
10	Sarcasm	12	6.3	3.5	14	3.3	1.2	6	5.5	5.8
11	Sincere	9	7.1	2.9	3	7.7	1.2	13	3.7	2.8
12	Sarcasm	2	8.4	1.6	4	7.3	1.2	2	3.5	2.8
13	Sarcasm	13	6.0	3.6	12	4.3	2.1	11	5.5	4.4
14	Sincere	11	6.4	3.0	11	4.7	3.1	10	5.4	3.8
15	Sarcasm	6	7.4	2.1	6	6.7	0.6	9	3.8	3.0

Table SIII: Ranking of items from Part 3 of N-TASIT, based on qualitative assessments

Notes: SD: Standard deviation; TBI: traumatic brain injury

		Total			Clinical experts			TBI individuals		
Item nr	Sarcasm/ Lie	Rank	Mean score	SD	Rank	Mean score	SD	Rank	Mean score	SD
1	Lie	7	7.6	2.3	9	6.0	0.0	5	8.8	2.5
2	Sarcasm	2	8.3	1.3	3	7.3	0.6	1	9.0	1.2
3	Lie	10	6.7	3.4	13	4.3	2.3	7	8.5	3.0
4	Sarcasm	16	5.0	3.0	15	3.3	1.2	14	6.3	3.5
5	Sarcasm	14	5.9	4.0	16	3.0	1.7	10	8.0	4.0
6	Lie	8	7.4	2.7	10	6.0	1.7	8	8.5	3.0
7	Lie	5	7.9	2.3	4	7.0	1.0	9	8.5	3.0
8	Sarcasm	13	6.1	3.0	11	6.0	2.0	15	6.3	3.9
9	Sarcasm	3	8.2	2.1	5	7.0	0.0	6	8.8	2.5
10	Sarcasm	4	8.0	2.0	6	6.7	1.2	2	9.0	2.0
11	Lie	1	8.7	1.5	1	8.3	0.6	3	9.0	2.0
12	Sarcasm	15	5.7	3.3	12	4.7	2.1	12	6.5	4.0
13	Lie	9	7.0	3.0	2	7.7	0.6	13	6.5	4.1
14	Sarcasm	11	6.7	3.0	14	4.0	0.0	11	8.0	2.8
15	Lie	6	7.9	2.1	7	6.3	1.2	4	9.0	2.0
16	Lie	12	6.3	3.0	8	6.3	2.1	16	6.3	3.9

Item elimination – Part 1

Table SIV: 1st round of item reduction results for N-TASIT Part 1

Notes: CFA: Confirmatory Factor Analysis; PBC: Point Biserial Correlation. Removed items in bold type

	Rasch analysis			CFA
**Item**	**Infit**	**Outfit**	**PBC**	**Factor loading**
Item 1	1.02	1.03	0.13	0.20
**Item 2**	**1.05**	**1.16**	**0.02**	**-0.02**
Item 3	1.00	0.96	0.21	0.51
Item 4	1.03	0.97	0.13	0.29
Item 5	0.94	0.87	0.22	0.27
**Item 6**	**0.85**	**0.47**	**0.35**	**0.94**
**Item 7**	**1.13**	**1.26**	**0.01**	**0.10**
Item 8	0.97	0.77	0.05	0.22
Item 10	0.96	0.78	0.18	0.34
Item 11	0.95	0.73	0.12	0.22
Item 12	0.95	0.84	0.12	0.30
Item 13	0.90	0.87	0.33	0.64
Item 14	0.94	0.87	0.19	0.45
Item 15	0.88	0.53	0.26	0.50
**Item 16**	**1.02**	**1.44**	**-0.08**	**0.11**
Item 17	0.94	0.87	0.18	0.23
Item 18	1.06	1.09	0.13	0.18
Item 19	0.99	1.02	0.21	0.54
Item 20	1.01	0.96	0.16	0.28
**Item 21**	**1.14**	**1.17**	**0.02**	**0.05**
Item 22	0.93	0.71	0.20	0.53
Item 23	0.93	0.63	0.19	0.29
**Item 24**	**1.01**	**0.88**	**0.07**	**0.08**
Item 25	0.90	0.59	0.24	0.44
Item 26	1.00	0.91	0.08	0.57
Item 27	1.05	1.24	0.10	0.16
Item 28	0.93	0.59	0.17	0.73

Table SV: 2nd round of item reduction results for N-TASIT Part 1

Notes: CFA: Confirmatory Factor Analysis; PBC: Point Biserial Correlation. Removed items in bold type.

	Rasch analysis			CFA
**Item**	**Infit**	**Outfit**	**PBC**	**Factor loading**
**Item 1**	**1.05**	**1.10**	**0.13**	**0.16**
Item 3	1.05	1.10	0.19	0.31
Item 4	1.08	1.12	0.10	0.34
Item 5	0.93	0.99	0.25	0.28
Item 8	0.96	0.88	0.04	0.24
Item 10	0.96	0.84	0.19	0.36
Item 11	0.97	0.82	0.07	0.27
Item 12	0.93	0.75	0.14	0.33
Item 13	0.92	0.84	0.32	0.58
Item 14	0.92	0.75	0.24	0.49
Item 15	0.89	0.53	0.23	0.48
Item 17	0.93	0.78	0.19	0.38
**Item 18**	**1.13**	**1.11**	**0.10**	**0.13**
Item 19	0.97	0.99	0.26	0.53
Item 20	1.05	1.11	0.16	0.27
Item 22	0.92	0.80	0.19	0.56
Item 23	0.93	0.64	0.18	0.37
Item 25	0.91	0.59	0.21	0.37
Item 26	0.99	0.81	0.12	0.63
**Item 27**	**1.10**	**1.25**	**0.09**	**0.15**
Item 28	0.92	0.59	0.17	0.78

Table SVI: 3rd round of item reduction results for N-TASIT Part 1

Notes: CFA: Confirmatory Factor Analysis; PBC: Point Biserial Correlation. Removed items in bold type.

	Rasch analysis			CFA
**Item**	**Infit**	**Outfit**	**PBC**	**Factor loading**
Item 3	1.10	1.14	0.18	0.30
Item 4	1.08	1.09	0.16	0.38
Item 5	1.01	1.16	0.16	0.22
**Item 8**	**1.00**	**1.69**	**-0.05**	**0.20**
Item 10	0.95	0.96	0.23	0.38
**Item 11**	**0.98**	**0.85**	**0.06**	**0.27**
Item 12	0.95	1.22	0.10	0.29
Item 13	0.94	0.98	0.32	0.58
Item 14	0.93	0.75	0.24	0.46
Item 15	0.88	0.56	0.22	0.45
Item 17	0.95	0.78	0.17	0.35
Item 19	0.92	0.92	0.34	0.56
Item 20	1.08	1.15	0.18	0.27
Item 22	0.90	0.73	0.23	0.60
Item 23	0.91	0.62	0.20	0.36
Item 25	0.94	0.70	0.16	0.34
Item 26	0.99	0.84	0.14	0.65
Item 28	0.89	0.57	0.19	0.82

Table SVII: 4th round of item reduction results for N-TASIT Part 1

Notes: CFA: Confirmatory Factor Analysis; PBC: Point Biserial Correlation.
Removed item in bold type.

	Rasch analysis			CFA
**Item**	**Infit**	**Outfit**	**PBC**	**Factor loading**
Item 3	1.11	1.15	0.18	0.30
Item 4	1.10	1.11	0.16	0.40
Item 5	1.02	1.18	0.16	0.22
Item 10	0.96	1.00	0.22	0.38
Item 12	0.94	1.20	0.11	0.27
Item 13	0.94	0.97	0.32	0.60
Item 14	0.92	0.73	0.27	0.50
Item 15	0.87	0.54	0.23	0.44
Item 17	0.97	0.87	0.16	0.32
Item 19	0.91	0.91	0.36	0.58
Item 20	1.09	1.16	0.18	0.28
Item 22	0.89	0.71	0.25	0.63
Item 23	0.90	0.59	0.22	0.35
Item 25	0.93	0.67	0.18	0.34
**Item 26**	**1.03**	**0.94**	**0.10**	**0.61**
Item 28	0.91	0.64	0.16	0.80

Table SVIII: 5th round of item reduction results for N-TASIT Part 1

Notes: CFA: Confirmatory Factor Analysis; PBC: Point Biserial Correlation. Removed items in bold type

	**Rasch analysis**			**CFA**
**Item**	**Infit**	**Outfit**	**PBC**	**Factor loading**
**Item 3**	**1.12**	**1.15**	**0.16**	**0.29**
Item 4	1.08	1.07	0.18	0.43
Item 5	1.01	1.14	0.17	0.34
Item 10	0.96	0.97	0.23	0.37
Item 12	0.92	1.15	0.13	0.35
Item 13	0.96	0.99	0.31	0.58
Item 14	0.90	0.71	0.28	0.59
Item 15	0.85	0.50	0.25	0.65
Item 17	0.96	0.89	0.17	0.38
Item 19	0.94	0.97	0.33	0.54
Item 20	1.07	1.12	0.20	0.36
Item 22	0.88	0.68	0.26	0.63
Item 23	0.90	0.58	0.22	0.46
Item 25	0.91	0.64	0.20	0.49
**Item 28**	**0.95**	**0.88**	**0.08**	**0.36**

Table SIX: 6th round of item reduction results for N-TASIT Part 1
Notes: CFA: Confirmatory Factor Analysis; PBC: Point Biserial Correlation.
Removed item in bold type

	Rasch analysis			CFA
**Item**	**Infit**	**Outfit**	**PBC**	**Factor loading**
Item 4	1.06	1.05	0.21	0.47
Item 5	1.01	1.05	0.20	0.40
Item 10	0.98	1.01	0.22	0.37
Item 12	0.91	1.07	0.16	0.36
Item 13	0.97	0.99	0.31	0.55
Item 14	0.92	0.89	0.26	0.57
Item 15	0.85	0.50	0.25	0.65
Item 17	0.96	1.17	0.17	0.39
Item 19	0.98	1.04	0.28	0.49
**Item 20**	**1.12**	**1.18**	**0.18**	**0.34**
Item 22	0.88	0.62	0.28	0.66
Item 23	0.89	0.56	0.24	0.48
Item 25	0.93	0.68	0.19	0.48

Table SX: 7th round of item reduction results for N-TASIT Part 1

Note: CFA: Confirmatory Factor Analysis; PBC: Point Biserial Correlation

	Rasch analysis			CFA
**Item**	**Infit**	**Outfit**	**PBC**	**Factor loading**
Item 4	1.09	1.11	0.21	0.49
Item 5	1.01	1.02	0.21	0.42
Item 10	0.98	0.98	0.22	0.39
Item 12	0.88	0.98	0.19	0.40
Item 13	0.99	0.99	0.31	0.56
Item 14	0.97	0.91	0.22	0.52
Item 15	0.86	0.59	0.23	0.61
Item 17	0.95	1.15	0.18	0.43
Item 19	1.05	1.12	0.25	0.46
Item 22	0.87	0.61	0.29	0.67
Item 23	0.87	0.55	0.26	0.49
Item 25	0.94	0.66	0.18	0.45

Item elimination – Part 2

Table SXI: 1st round of item reduction results for N-TASIT Part 2, Sarcasm

Notes: CFA: Confirmatory Factor Analysis; PBC: Point Biserial Correlation.
Removed items in bold type

	Rasch analysis			CFA
**Item**	**Infit**	**Outfit**	**PBC**	**Factor loading**
Item 2	0.82	0.72	0.60	0.84
Item 3	0.94	0.84	0.53	0.76
Item 5	0.99	1.13	0.44	0.70
**Item 6**	**0.97**	**2.18**	**0.32**	**0.61**
**Item 8**	**0.95**	**0.48**	**0.36**	**0.73**
Item 9	0.86	0.70	0.55	0.81
Item 10	0.91	0.86	0.52	0.76
Item 13	0.80	0.72	0.58	0.85
Item 15	1.07	1.32	0.43	0.64

Table SXII: 2nd round of item reduction results for N-TASIT Part 2, Sarcasm

Note: CFA: Confirmatory Factor Analysis; PBC: Point Biserial Correlation

	Rasch analysis			CFA
**Item**	**Infit**	**Outfit**	**PBC**	**Factor loading**
Item 2	0.82	0.75	0.61	0.85
Item 3	0.94	0.88	0.55	0.77
Item 5	1.02	1.20	0.42	0.70
Item 9	0.92	0.77	0.56	0.82
Item 10	0.92	0.87	0.51	0.77
Item 13	0.80	0.75	0.58	0.85
Item 15	1.15	1.35	0.41	0.62

Table SXIII: 1st round of item reduction results for N-TASIT Part 2, Sincere

Notes: CFA: Confirmatory Factor Analysis; PBC: Point Biserial Correlation. Removed item in bold type

	Rasch analysis			CFA
**Item**	**Infit**	**Outfit**	**PBC**	**Factor loading**
Item 1	0.91	0.85	0.37	0.63
Item 4	0.81	0.85	0.35	0.65
**Item 7**	**1.29**	**1.29**	**0.03**	0.02
Item 11	0.83	0.74	0.32	0.81
Item 14	0.98	0.92	0.32	0.60

Table SXIV: 2nd round of item reduction results for N-TASIT Part 2, Sincere

Note: CFA: Confirmatory Factor Analysis; PBC: Point Biserial Correlation

	Rasch analysis			CFA
**Item**	**Infit**	**Outfit**	**PBC**	**Factor loading**
Item 1	0.95	0.97	0.35	0.63
Item 4	0.94	0.97	0.35	0.65
Item 11	0.85	0.70	0.41	0.82
Item 14	0.98	0.94	0.35	0.60

Item elimination – Part 3

Table SXV: 1st round of item reduction results for N-TASIT Part 3, Lies

Notes: CFA: Confirmatory Factor Analysis; PBC: Point Biserial Correlation.
Removed items in bold type

	Rasch analysis			CFA
**Item**	**Infit**	**Outfit**	**PBC**	**Factor loading**
Item 1	1.12	1.16	0.72	0.44
Item 3	0.90	0.89	0.53	0.77
Item 6	0.74	0.72	0.44	0.87
**Item 7**	**1.20**	**1.59**	**0.83**	**0.27**
**Item 11**	**0.66**	**0.34**	**0.90**	**0.93**
Item 13	0.86	0.73	0.82	0.69
Item 15	0.83	0.93	0.79	0.72
Item 16	1.09	1.14	0.60	0.56

Table SXVI: 2nd round of item reduction results for N-TASIT Part 3, Lies

Note: CFA: Confirmatory Factor Analysis; PBC: Point Biserial Correlation

	Rasch analysis			CFA
**Item**	**Infit**	**Outfit**	**PBC**	**Factor loading**
Item 1	1.14	1.19	0.26	0.44
Item 3	0.90	0.87	0.45	0.79
Item 6	0.71	0.68	0.54	0.95
Item 13	0.85	0.87	0.36	0.62
Item 15	0.86	0.94	0.38	0.61
Item 16	1.11	1.21	0.31	0.49

Table SXVII: 1st round of item reduction results for N-TASIT Part 3, Sarcasm

Notes: CFA: Confirmatory Factor Analysis; PBC: Point Biserial Correlation.
Removed item in bold type

	Rasch analysis			CFA
**Item**	**Infit**	**Outfit**	**PBC**	**Factor loading**
Item 2	0.75	0.63	0.32	0.72
Item 4	0.86	0.76	0.44	0.75
**Item 5**	**1.36**	**1.41**	**0.13**	**0.18**
Item 8	1.02	1.04	0.35	0.50
Item 9	1.08	0.87	0.30	0.54
Item 10	0.83	0.81	0.50	0.85
Item 12	1.00	0.98	0.38	0.53
Item 14	0.80	0.74	0.52	0.87

Table SXVIII: 2nd round of item reduction results for N-TASIT Part 3, Sarcasm

Note: CFA: Confirmatory Factor Analysis; PBC: Point Biserial Correlation

	Rasch analysis			CFA
**Item**	**Infit**	**Outfit**	**PBC**	**Factor loading**
Item 2	0.71	0.62	0.32	0.72
Item 4	0.87	0.74	0.48	0.76
Item 8	1.15	1.29	0.32	0.49
Item 9	1.11	1.08	0.32	0.54
Item 10	0.83	0.77	0.54	0.86
Item 12	1.15	1.07	0.36	0.52
Item 14	0.82	0.76	0.54	0.87

Table SXIX: The convergent and discriminant validity of the short version versus the full version of N-TASIT

Note: BRI: Behavior Regulation Index; BRIEF: Behavior Rating Inventory of Executive Function - Adult version; CWIT: Color Word Interference Test; D-KEFS: Delis-Kaplan Executive Function System; ERT: Emotional recognition task; FSS: Fatigue Severity Scale; GAD7: Global Anxiety Disorder; GEC: Global Executive Composite; IRI: Interpersonal Reactivity Index; MI: Metacognition Index; N-TASIT: The Norwegian Awareness of Inference Test; PHQ9: Patient Hospital Questionnaire 9 Items; rho: Spearman rank correlations; WAIS IV: Wechsler Adult Intelligence Scale IV. Non-significant associations in the short version that were significant in the full version are highlighted in bold type.

	Short version								Full version							
	Total		Part 1		Part 2		Part 3		Total		Part 1		Part 2		Part 3	
Test/Questionnaire	rho	p	rho	p	rho	p	rho	p	rho	p	rho	p	rho	p	rho	p
Convergent validity																
Hinting Task	0.37	<0.001	0.20	0.049	0.32	0.001	0.29	0.004	0.37	<0.001	0.26	0.010	0.34	0.001	0.32	0.001
ERT: Total score	0.50	<0.001	0.46	<0.001	0.38	<0.001	0.46	<0.001	0.50	<0.001	0.52	<0.001	0.42	<0.001	0.43	<0.001
IRI: Perspective Taking	**0.14**	**0.184**	0.09	0.365	0.14	0.173	**0.12**	**0.236**	0.20	0.048	0.17	0.104	0.12	0.262	0.24	0.018
IRI: Emotional Concern	-0.08	0.422	-0.03	0.747	-0.02	0.821	-0.13	0.201	-0.07	0.524	-0.05	0.614	-0.10	0.346	-0.03	0.744
Discriminant validity																
WAIS IV: Similarities	0.39	<0.001	0.42	<0.001	0.26	0.009	0.34	0.001	0.41	<0.001	0.41	<0.001	0.30	0.002	0.38	<0.001
WAIS IV: Matrices	0.31	0.002	0.24	0.018	0.31	0.002	**0.18**	**0.069**	0.31	0.001	0.25	0.013	0.37	<0.001	0.21	0.040
WAIS IV: Coding	0.43	<0.001	0.28	0.005	0.37	<0.001	0.38	<0.001	0.46	<0.001	0.42	<0.001	0.43	<0.001	0.37	<0.001
WAIS IV: Digit Span	0.24	0.014	**0.19**	**0.065**	0.23	0.020	0.20	0.047	0.28	0.004	0.28	0.005	0.30	0.002	0.17	0.083
D-KEFS: CWIT 3	0.31	0.002	**0.18**	**0.078**	0.28	0.004	0.28	0.006	0.35	<0.001	0.29	0.003	0.34	<0.001	0.28	0.005
D-KEFS: Trails 4	0.23	0.022	0.08	0.405	0.27	0.007	0.14	0.172	0.23	0.021	0.12	0.221	0.35	<0.001	0.09	0.395
BRIEF: BRI	-0.08	0.411	0.05	0.597	-0.17	0.103	-0.02	0.810	-0.09	0.403	-0.09	0.361	-0.11	0.260	-0.03	0.801
BRIEF: MI	0.07	0.478	0.16	0.112	-0.07	0.516	0.15	0.142	0.06	0.535	0.00	0.995	0.00	0.981	0.14	0.159
BRIEF: GEC	0.02	0.858	0.14	0.171	-0.10	0.316	0.08	0.414	0.01	0.937	-0.03	0.744	-0.04	0.687	0.07	0.467
FSS	0.08	0.418	0.17	0.105	0.05	0.626	0.03	0.779	0.08	0.417	0.12	0.249	0.07	0.485	0.06	0.556
PHQ9 & GAD7	0.08	0.465	0.09	0.391	0.07	0.500	0.03	0.750	0.06	0.539	-0.02	0.828	0.05	0.605	0.10	0.353
